# Outpatient visits before and after Lyme disease diagnosis in a Maryland employer-based health plan

**DOI:** 10.1186/s12913-023-09909-3

**Published:** 2023-08-29

**Authors:** Alison W. Rebman, Ting Yang, Lin Wang, Jill A. Marsteller, Shannon M. E. Murphy, Maria Uriyo, John N. Aucott

**Affiliations:** 1 Lyme Disease Research Center, Division of Rheumatology, Department of Medicine, Johns Hopkins University School of Medicine, Baltimore, MD USA; 2 Johns Hopkins HealthCare LLC, Glen Burnie, MD USA; 3 Department of Health Policy and Management, Bloomberg School of Public Health, Johns Hopkins University, Baltimore, MD USA; 4 Department of Health Administration and Policy, George Mason University, Fairfax, VA USA

**Keywords:** Lyme disease, Tick-borne diseases, Post-treatment Lyme disease, Claims analysis, Maryland

## Abstract

**Background:**

Insurance claims data have been used to inform an understanding of Lyme disease epidemiology and cost of care, however few such studies have incorporated post-treatment symptoms following diagnosis. Using longitudinal data from a private, employer-based health plan in an endemic US state, we compared outpatient care utilization pre- and post-Lyme disease diagnosis. We hypothesized that utilization would be higher in the post-diagnosis period, and that temporal trends would differ by age and gender.

**Methods:**

Members with Lyme disease were required to have both a corresponding ICD-9 code and a fill of an antibiotic indicated for treatment of the infection within 30 days of diagnosis. A 2-year ‘pre- diagnosis’ period and a 2-year ‘post-diagnosis period’ were centered around the diagnosis month. Lyme disease-relevant outpatient care visits were defined as specific primary care, specialty care, or urgent care visits. Descriptive statistics examined visits during these pre- and post-diagnosis periods, and the association between these periods and the number of visits was explored using generalized linear mixed effects models adjusting for age, season of the year, and gender.

**Results:**

The rate of outpatient visits increased 26% from the pre to the post-Lyme disease diagnosis periods among our 317-member sample (rate ratio = 1.26 [1.18, 1.36], *p* < 0.001). Descriptively, care utilization increases appeared to persist across months in the post-diagnosis period. Women’s care utilization increased by 36% (1.36 [1.24, 1.50], *p* < 0.001), a significantly higher increase than the 14% increase found among men (1.14 [1.02, 1.27], *p* = 0.017). This gender difference was mainly driven by adult members. We found a borderline significant 17% increase in visits for children < 18 years, (1.17 [0.99, 1.38], *p* = 0.068), and a 31% increase for adults ≥ 18 years (1.31 [1.21, 1.42], *p* < 0.001).

**Conclusions:**

Although modest at the population level, the statistically significant increases in post-Lyme diagnosis outpatient care we observed were persistent and unevenly distributed across demographic and place of service categories. As Lyme disease cases continue to grow, so will the cumulative prevalence of persistent symptoms after treatment. Therefore, it will be important to confirm these findings and understand their significance for care utilization and cost, particularly against the backdrop of other post-acute infectious syndromes.

**Supplementary Information:**

The online version contains supplementary material available at 10.1186/s12913-023-09909-3.

## Background

Lyme disease (LD) is a tick-borne infection caused by various genospecies of the bacteria *Borrelia burgdorferi* sensu lato complex [1]. The Centers for Disease Control and Prevention (CDC) has estimated that 476,000 patients are diagnosed and treated for LD in the US annually, with increasing cases in recent years due to geographic expansion of the tick vector and shifting land use patterns [2, 3]. LD incidence has historically been strongly geographically determined, with cases primarily concentrated in the northeastern, mid-Atlantic, upper Midwestern, and Pacific coast states [4]. Surveillance for LD is conducted through passive reporting in the US and is known to reflect significant underreporting of cases [5, 6]. The use of insurance claims data in LD has limitations of generalizability and specificity, however it has been shown to be useful in informing a broader understanding of LD prevalence and disease trends, particularly in states with high LD incidence [7].

Even in the context of appropriate and timely antibiotic treatment of early LD, a subset of patients develop persistent symptoms such as fatigue, musculoskeletal pain, and cognitive difficulties [8]. When patients subsequently meet criteria for a specific, research-based case definition, these symptoms can additionally be referred to as post-treatment Lyme disease (PTLD). There are no FDA-approved treatment options for these persistent symptoms, which can last for months to years with substantial impacts on health-related quality of life [9–11]. Similar to other post-acute infection syndromes, including long COVID, much remains unknown or disputed about illness prevalence, severity, risk factors, and pathophysiology [8, 12]. A recent modeling study estimated the cumulative prevalence of PTLD at 1–2 million people in the US alone [13].

Prior studies have examined care utilization and costs associated with a LD diagnosis [14]. However, the burden of PTLD at the population level has been difficult to quantify, therefore few studies have examined these trends temporally or incorporated the potential additional impact of persistent symptoms in the months or years following diagnosis in their analyses. One such large-scale study of national insurance claims found 87% more outpatient visits and almost $3,000 higher health care costs among patients in the 12-month period following LD diagnosis compared to matched controls, amounting to upwards of $1 billion per year in direct medical costs [15].

In the current retrospective study, we drew upon longitudinal claims data from a large, employer-based health insurance plan in a Lyme-endemic US state to examine trends in outpatient care utilization after treatment of diagnosed LD. We used members’ own pre-LD diagnosis period as comparison. We hypothesized that the number of outpatient visits in members’ post-LD diagnosis period would be higher than their pre-LD diagnosis period, and that temporal trends in increased utilization would differ by members’ age and gender. Specifically, we hypothesized any increases in utilization would be more pronounced for women than for men, and for adults than for children, given previously observed trends in prevalence of persistent symptoms following LD ﻿[8, 16–20].

## Methods

### Study sample

Our initial sample included retrospective person-month level data from members of Johns Hopkins Employer Health Programs (EHP), a private, employer-based program. All residents of Maryland enrolled at any time over a 7-year period (July 2004-June 2011) with a diagnosis code of LD (as described below) were included. For each member, a 48-month (4-year) study window was created around the LD diagnosis month, with months − 24 to -1 representing the ‘pre-LD diagnosis’ period, month 0 representing the month of LD diagnosis, and months 1 to 23 representing the ‘post-LD diagnosis’ period. Members were not required to contribute continuous data at each of these months, therefore each member contributed a varying number of months to the final data set. While we assumed that any missing months would be randomly distributed relative to members’ LD diagnosis month, we also performed a sensitivity analysis only among those with 48 months of continuous membership to ensure that this attribute of the data did not substantively affect our results.

### Lyme disease diagnoses

Diagnoses of LD were defined as detailed by the authors in a previous study [21]. Briefly, the first paid medical professional claim with International Classification of Diseases, Ninth Revision, Clinical Modification (ICD-9), code 088.81 was identified [22]. This diagnosis code encompasses all stages of Lyme disease. Any members with a diagnosis 1 year prior to the study period (July 1, 2003 through June 30, 2004) were excluded from the sample to reduce spillover of previously incident cases. To increase diagnostic specificity in the current study, we also required a fill within 30 days of an antibiotic indicated for treatment of Lyme disease. Doxycycline, tetracycline, cefuroxime, amoxicillin, ampicillin, ceftriaxone, amoxicillin/clavulanate potassium, and azithromycin (if given at least a 14 days’ supply) were considered to be indicated antibiotics.

### Variables of interest

LD-relevant outpatient health care visits were defined as any of the following; (a) *primary care* visits (a provider with one of the following specialties: general practice, family practice, family medicine, pediatrics, internal medicine without other specialties, and nurse practitioner); (b) *specialty care* visits (a provider with one of the following as their primary practice: infectious diseases, neurology, ophthalmology, otolaryngology, cardiology, rheumatology, orthopedics, physical therapy, or mental health); and (c) *urgent care* visits (an urgent care or emergency department resulting in either treat-and-release or hospitalization). As a measure of health-care utilization, a monthly sum of these three types of visits was generated as the outcome and used to calculate the rate of outpatient visits per person-month. Lyme disease diagnosis period (pre vs. post diagnosis month) was the primary predictor. Covariates of interest included gender, age, and season. Age was treated in the following ways, depending on the aims of the specific analysis: (a) as a continuous variable for descriptive purposes; (b) categorized into 10-year increments and centered for regression analyses; and (c) dichotomized by members’ age in the last available month for each member (< 18 years vs. ≥ 18 years) to examine differences in children compared to adults (i.e. “adult status”). Similarly, season was treated in the following ways, depending on the aims of the specific analysis: (a) “season of the year” (e.g. spring, summer, fall, winter), and (b) “LD incidence season” based on the monthly distribution of confirmed LD cases reported to the CDC [23]. We considered low Lyme disease season to be December – March, medium Lyme disease season to be April, May, September – November, and high Lyme disease season to be June – August.

### Statistical analyses

We first removed any members with an outlying total number of outpatient visits in a given month, as determined by both (a) the maximum, and (b) the magnitude of the difference between the maximum and the second maximum values for each member across all months. This represents members with an unusually high number of visits in only one month, skewing the overall data for that month. Next, descriptive statistics were calculated to summarize members’ characteristics during the month of their LD diagnosis, and to display outpatient visits during the pre and post-LD diagnosis periods.

We then explored the association between the number of all LD-relevant outpatient visits and LD diagnosis period (e.g. pre vs. post) through multivariate analyses adjusting for age, season of the year, and gender, as we hypothesized that these factors may independently affect care utilization. We used generalized linear mixed effects models with a log-link and a negative binomial variance for the monthly visit count. A negative binomial distribution was selected over a Poisson distribution to account for over-dispersion. To account for the correlation of monthly visits contributed by the same member over time, member was included as a random intercept. Based on existing literature, we also hypothesized that there would be gender and adult status effects on the relationship between number of outpatient visits and LD diagnosis period. Therefore, we also studied models that included an interaction term between gender and LD diagnosis period. Due to collinearity between the age and adult status variables, adult status effects could not be studied with interaction models. As a result, this association was explored in models fit on children and adults separately.

All reported *p*-values are 2-sided. All statistical analyses and graphs were generated using R, version 4.2.0 (R Foundation for Statistical Computing, Vienna, Austria).

## Results

**Fig. 1:**
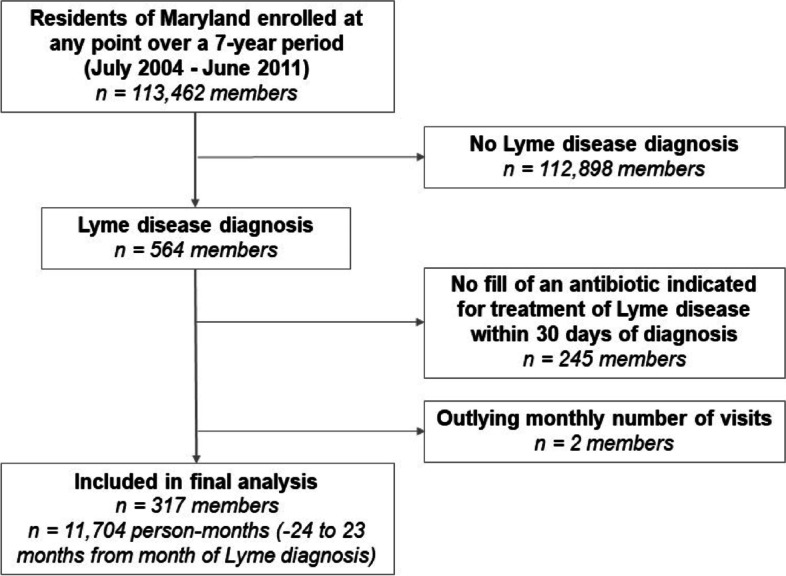
Study population and sample size included in final analyses

Our initial sample included 113,462 EHP members, 564 of whom had a LD diagnosis, for an average of 80.57 LD diagnoses annually. After accounting for outliers (0.4%) and those without an indicated antibiotic fill (43.4%), a final sample of 317 members were included in the analysis representing 11,704 person-months (Fig. 1).

Members contributed an average of 36.92 (standard deviation 10.69, range 6–48) months of follow-up within the 48-month study time frame. Members contributed an average of 18.18 months (standard deviation 7.10, range 1–23) in the post-LD diagnosis period, and 18.66 months (standard deviation: 7.57, range 1–24) in the pre-LD diagnosis period. The demographic characteristics of this sample, as well as the LD incidence level in the month of their diagnosis, and the place of service of their diagnosis, are shown in Table 1.

**Table 1:** Characteristics of 317 private health insurance plan members during the month of their Lyme disease diagnosis. The N (%) or median [IQR] (range) are presented for each

	Members (*n* = 317)
Age (years)	39.60 [19.50, 50.70] (0.40, 73.00)
Adult status	
< 18 years	75 (23.7%)
≥ 18 years	242 (76.3%)
Gender	
Men	148 (46.7%)
Women	169 (53.3%)
Metropolitan Area of Residence^a^	
Baltimore	291/316 (92.1%)
Other	25/316 (7.9%)
Lyme Disease Incidence Season	
Low (December – March)	30 (9.5%)
Medium (April, May, September – November)	99 (31.2%)
High (June – August)	188 (59.3%)
Diagnosis Place of Service	
Office visit	251 (79.2%)
Urgent care visit	27 (8.5%)
Inpatient visit	18 (5.7%)
Emergency room visit	14 (4.4%)
Other outpatient visit	7 (2.2%)

^a^ *Based on Metropolitan Statistical Areas defined by the United States Office of Management and Budget *[24].* One member missing area of residence at the time of LD diagnosis, no other missing data*

In our sample, 59.3% were diagnosed during typical summer months. Although our overall sample had a slightly higher proportion of women (53.3%), a higher proportion of children < 18 years of age (50/75, 66.7%) were boys, consistent with surveillance data in which LD has a higher incidence among young boys compared to girls.

**Fig. 2:**
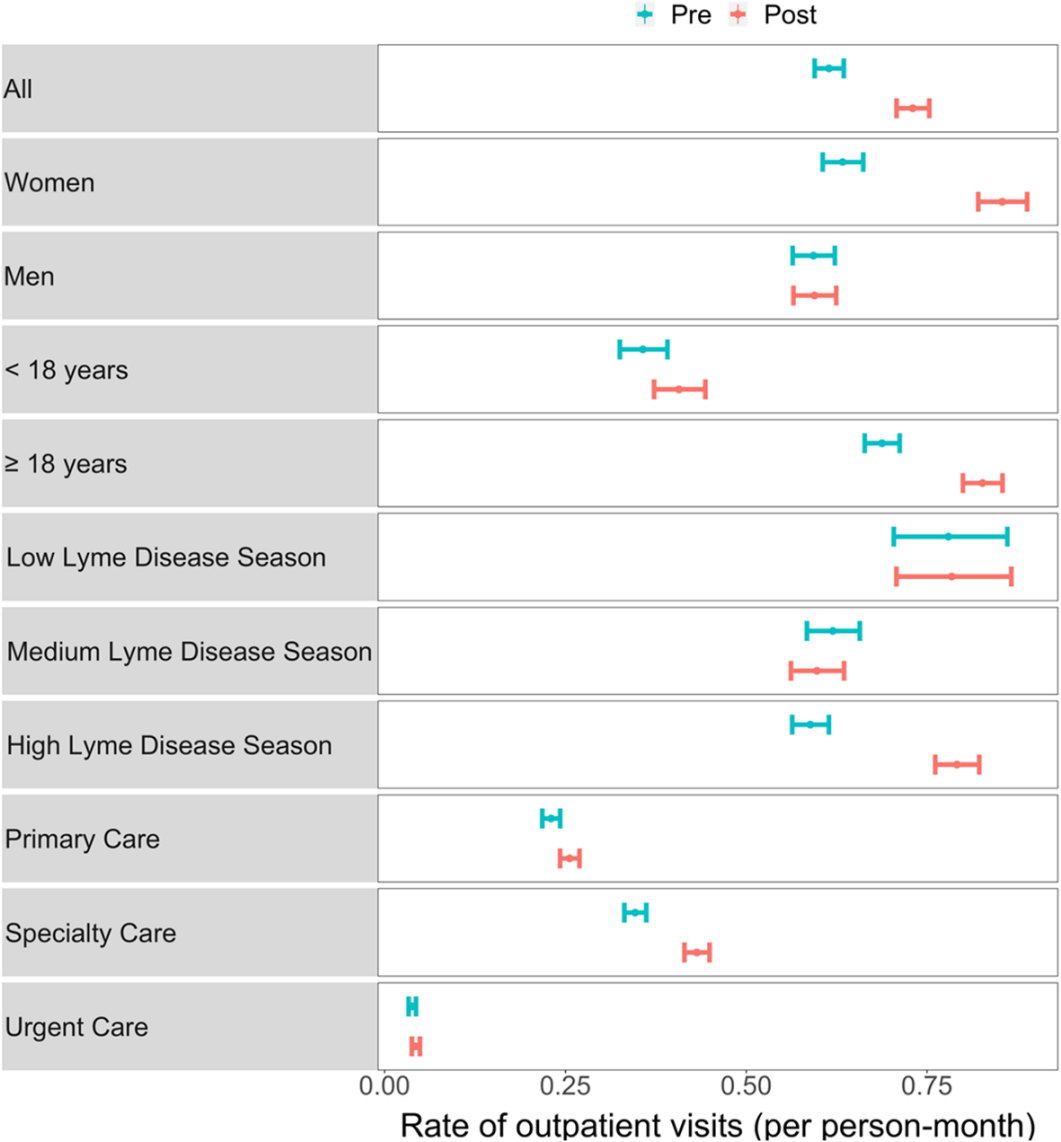
Unadjusted rate and 95% confidence interval of Lyme disease relevant outpatient visits stratified by pre (24 months prior) vs. post (23 months after) Lyme disease diagnosis period

Figure 2 depicts the unadjusted rate of LD-relevant outpatient visits with all months combined within the pre and post-diagnosis periods.

**Fig. 3:**
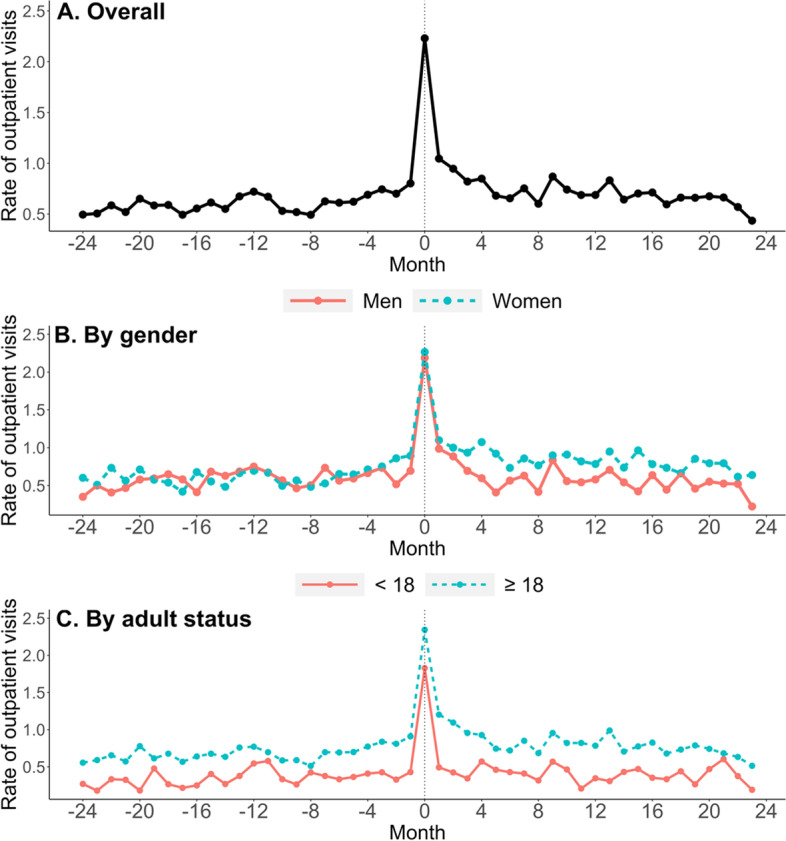
Unadjusted rate of Lyme disease-relevant outpatient visits in the 24 months before LD diagnosis (-24 to -1), the month of Lyme disease diagnosis (0), and the 23 months after Lyme disease diagnosis (1 to 23). Note that for monthly data, rate of outpatient visits per person-month is equivalent to the average number of visits per person per month

Increases in care utilization during the post-diagnosis period were observed in the sample as a whole (change from pre to post: +0.12 visits per person-month), and among women (+ 0.22), adults (+ 0.14), and those diagnosed during high LD season months (+ 0.20). Increases were also more marked in specialty care (+ 0.09) compared to primary or urgent care. Outpatient care utilization was high in both the pre- and post-diagnosis periods for those diagnosed during low LD season months. When the unadjusted rate of outpatient visits is instead depicted longitudinally at each month rather than collapsed into periods, increases in the number of outpatient visits appear more frequently in the months immediately before and after the LD diagnosis (Fig. 3).

**Fig. 4:**
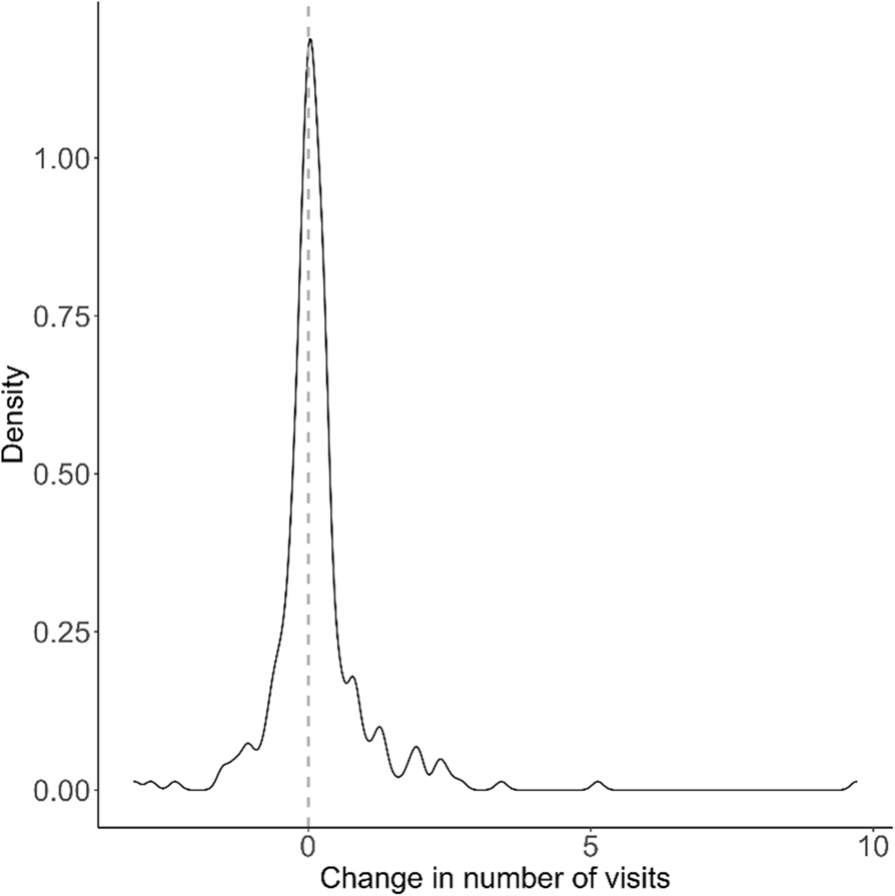
Density plot of member-level change in the average number of Lyme disease-relevant outpatient care visits per month from the pre- to the post-Lyme disease diagnosis periods

However, particularly among women, increases in outpatient visits appear sustained in the post-diagnosis period out to a year and beyond. Figure 4 shows a right-skewed distribution of the difference between the pre-and post-LD diagnosis period, where the median increase in member-level average monthly visits was 0.06 (interquartile range [IQR]: -0.15, 0.31; range: -3.08, 9.70), significantly different from 0 (*p* = 0.001).

After controlling for gender, age, and season of the year, the rate of LD-relevant outpatient visits increased 26% from the pre to the post-LD diagnosis periods (Table 2, Model 1: rate ratio [RR] = 1.26 [1.18, 1.36], *p* < 0.001).

**Table 2:** Generalized linear mixed effects regression models with number of all Lyme disease-relevant outpatient care visits as the outcome. Models were run on the overall sample [1], as well as on adult [2] and children [3] strata only. Additional models [4, 5, and 6] were run with a gender and Lyme disease diagnosis period (pre vs. post) interaction term included

	*Overall Sample* *N*^*a*^*=317*		*Children Only* *N = 70*^*b*^		*Adults Only* *N = 247*^*b*^	
**Models with no interaction terms**	**Model 1**		**Model 2**		**Model 3**	
	**RR** ^**c**^	***p*** **-value**	**RR**	***p*** **-value**	**RR**	***p*** **-value**
Pre-Lyme disease diagnosis period	REF	REF	REF	REF	REF	REF
Post-Lyme disease diagnosis period	1.26 [1.18, 1.36]	< 0.001	1.17 [0.99, 1.38]	0.068	1.31 [1.21, 1.42]	< 0.001
Men	REF	REF	REF	REF	REF	REF
Women	1.34 [1.05, 1.70]	0.016	0.95 [0.66, 1.35]	0.764	1.44 [1.08, 1.92]	0.013
Age (10 years)	1.09 [1.02, 1.16]	0.007	0.70 [0.48, 1.02]	0.060	1.13 [1.01, 1.25]	0.029
Winter	REF	REF	REF	REF	REF	REF
Spring	1.08 [0.99, 1.19]	0.080	1.19 [0.97, 1.46]	0.099	1.07 [0.96, 1.18]	0.214
Summer	1.15 [1.05, 1.26]	0.002	1.03 [0.83, 1.28]	0.781	1.18 [1.06, 1.30]	0.002
Fall	1.06 [0.97, 1.16]	0.191	1.31 [1.07, 1.61]	0.008	1.01 [0.91, 1.12]	0.877
**Models with a gender interaction term**	**Model 4**		**Model 5**		**Model 6**	
	**RR**	***p*** **-value**	**RR**	***p*** **-value**	**RR**	***p*** **-value**
Pre-Lyme disease diagnosis period	REF	REF	REF	REF	REF	REF
Post-Lyme disease diagnosis period	1.14 [1.02, 1.27]	0.017	1.27 [1.03, 1.55]	0.023	1.13 [0.99, 1.28]	0.068
Men	REF	REF	REF	REF	REF	REF
Women	1.22 [0.95, 1.56]	0.118	1.06 [0.71, 1.56]	0.783	1.27 [0.94, 1.71]	0.119
Women: Post-Lyme disease diagnosis period (interaction)	1.20 [1.04, 1.38]	0.013	0.80 [0.58, 1.10]	0.169	1.27 [1.08, 1.49]	0.004
Age (10 years)	1.09 [1.02, 1.16]	0.006	0.69 [0.47, 1.01]	0.055	1.13 [1.01, 1.25]	0.026
Winter	REF	REF	REF	REF	REF	REF
Spring	1.08 [0.99, 1.19]	0.085	1.19 [0.97, 1.46]	0.097	1.06 [0.96, 1.18]	0.228
Summer	1.15 [1.05, 1.26]	0.002	1.03 [0.83, 1.28]	0.769	1.18 [1.06, 1.30]	0.002
Fall	1.06 [0.97, 1.16]	0.195	1.31 [1.08, 1.61]	0.008	1.01 [0.91, 1.12]	0.879

^a^*N *Number of unique members

^b^For multi-variate analyses, we defined adult status based on age at the last available month of data to keep adult status unique for each member. Therefore, the numbers of children and adults do not exactly match Table 1, in which adult status was defined based on age at the time of Lyme disease diagnosis; there were 5 members who were < 18 years of age (children) at the time of Lyme disease diagnosis who were subsequently ≥ 18 years of age (adults) when they contributed their last month of data

^c^*RR *rate ratio. All but the interaction terms are rate ratios. The interaction terms in Models 4, 5, and 6 are ratios of rate ratios

Although not statistically significant, there was a trend for women to have a higher rate of visits in the pre-LD diagnosis period compared to men (Model 4: RR = 1.22 [0.95, 1.56], *p* = 0.118). Furthermore, the rate of visits for women increased significantly more than that of men in the post-LD diagnosis period (Model 4 interaction: ratio of RRs: 1.20 [1.04, 1.38], *p* = 0.013). Specifically, care utilization by men increased by 14% (Model 4: RR = 1.14 [1.02, 1.27], *p* = 0.017), whereas care utilization by women increased by 36% (RR = 1.36 [1.24, 1.50], *p* < 0.001, calculated using Model 4 but with women as the reference group).

This difference by gender was mainly driven by adult members. Among children, boys had higher increases in the number of visits from the pre- to post-LD diagnosis periods than girls, though this difference between boys and girls was not statistically significant (Model 5: boys: 1.27 [1.03, 1.55], *p* = 0.023; girls: RR = 1.01 [0.77, 1.32], *p* = 0.937; interaction: ratio of RRs: 0.80 [0.58, 1.10], *p* = 0.169). By comparison, among adults, this gender difference was significant (Model 6: adult men: 1.13 [0.99, 1.28], *p* = 0.068; adult women: RR = 1.43 [1.29, 1.58], *p* < 0.001; interaction: ratio of RRs: 1.27 [1.08, 1.49], *p* = 0.004). Overall, we found a 17% increase in visits for children, although this was of borderline statistical significance (Model 2: RR = 1.17 [0.99, 1.38], *p* = 0.068), and a 31% increase for adults (Model 3: RR = 1.31 [1.21, 1.42], *p* < 0.001) from the pre to the post-LD diagnosis period. However, the difference in the increases between children and adults was not statistically significant (*p* = 0.234, manually calculated).

We performed a sensitivity analysis including only members who contributed continuous data at every month over the 48-month interval (*n* = 95) and the results were consistent with the primary analysis (regression results in Supplemental Table 1).

## Discussion

We conducted a retrospective, longitudinal pre-post study of claims data from members of a private health insurance plan largely residing in urban or peri-urban regions in Maryland. In the overall sample of 317 members with a LD diagnosis, we found a 26% increase in outpatient health care visits in the 2 years following the month of LD diagnosis compared to the 2 years before, after controlling for gender, age, and season of the year. Among adult members, this increase was significantly higher among women compared to men. Although we did not examine temporal trends in care utilization within the post-LD period statistically, these increases appear to arise in the months immediately prior to diagnosis and persist across months over 2 years beyond the immediate convalescent period. These results are similar to a prior study of national-level insurance claims which found an increase in outpatient visits in the 12 months following treatment for LD compared to matched controls [15]. The reason for the increases we observed in the months immediately prior to diagnosis are unknown, but they may reflect diagnostic uncertainty or misdiagnosis of the initial presentation.

Ultimately, we cannot be certain that the statistically significant outpatient claim increases we observed in the post-LD diagnosis period are due to persistent symptoms and not alternative factors. Nevertheless, we posit this as a reasonable explanation based on several aspects of our analysis. First, our findings remained significant in multivariate analyses controlling for gender, age, and season of the year, factors hypothesized to generally affect care utilization patterns. Second, the extended duration of individual, monthly data points on either side of the LD diagnosis helped to account for expected month-to-month variability and allowed us to generate a pre-morbid baseline of outpatient care patterns for comparison to the post-LD diagnosis period. Despite the fact that members were allowed to contribute a varying number of data points, sensitivity analyses among those with complete, continuous data also found similar trends. Lastly, our study period (2004–2011) occurred well before SARS-COV-2. While other historical events may have temporarily driven an increase in care visits, given our wide study window, the inclusion of members across multiple years, and the anchoring of diagnosis month seasonally throughout the year, we would hypothesize that these would be randomly distributed before and after LD diagnosis in our sample and would not affect comparisons between the pre and post-LD periods in a systematic way.

In the convalescent period following appropriate treatment for LD, approximately 10–20% of patients with early LD will meet criteria for a research case definition for PTLD which incorporates both symptoms and functional impact [25, 26]). Therefore, persistent symptoms will likely have an outsized effect on individual patients compared to what can be identified in the larger population diagnosed and treated for LD. Consequently, while the utilization increases we observed in the overall sample are statistically relevant, we would not expect them to be exceedingly large (approximately 0.12 increase in visits per month overall; 0.22 among women). The distribution of the magnitude of these utilization increases depicted in Fig. 4 shows a right-skewed tail that may be highlighting a subset of members with persistent symptoms.

Although the magnitude of the outpatient care increases we observed are relatively modest, they are likely clinically meaningful, particularly at the larger population level. They appeared most marked within specialty care, which may lead to new diagnostic testing and/or prescriptions thereby incurring greater costs. Furthermore, they appear to persist for longer than the immediate convalescent period, continuing to generate additional costs and reflect morbidity among those who remain ill for years to even decades [9, 27]. With the exception of the study by Adrion et al., [15] estimates of LD cost don’t typically consider the added impact of persistent symptoms. By comparing members to their own pre-LD diagnosis period, our findings further support a significant, population-level increase in utilization post-LD diagnosis which is likely distinct from expected levels of outpatient care utilization in the general population and should be considered in such analyses.

It is generally reported that utilization of health care services varies by sex/gender, [28]. We did note a trend for women to have a higher rate of visits in the baseline pre-LD diagnosis period than men, although it was not statistically significant. Regardless, in our data among adults, women had a significantly higher increase in outpatient visits in the post-LD diagnosis period compared to men. This is consistent with the observation that the risk of developing post-treatment symptoms may be increased among women, particularly in the broader clinical setting where specific research definitions for PTLD are not always applied [17]. Interestingly, we did not find a similar gender interaction among children as we did among adults. This could be due to a range of factors and is also left for future studies to address.

Overall, interpretation of our findings among patients under 18 years of age is less straightforward, but our data suggest a modest increase in outpatient visits among children in the post-LD diagnosis period. The 17% increase in visits we observed among children was of borderline statistical significance, however it was also statistically no different than the increase in visits seen among adults, which was of greater magnitude. Given that our sample size among children was much smaller than adults, it is possible that we lacked statistical power to detect this trend and a larger sample would be needed to properly evaluate differences in children compared to adults. While several previous studies have reported that the rate of persistent symptoms among children is low in appropriately and promptly treated patients, [18–20] direct comparison of this rate among adults and children is difficult and to our knowledge has not been reported.

There are several additional limitations to our study. There is inherently lower diagnostic specificity when relying on ICD codes, although we attempted to address this by requiring a relevant antibiotic fill within 30 days of a LD diagnosis. Notably, this criterion excluded over 40% of our initial sample. The relevance of this large subset of patients without a fill within 30 days is unknown. However, it may represent patients with a tentative LD diagnosis which was later ruled out, those given LD codes in error or at a date divorced from their actual diagnosis date, as well as those who experienced treatment delays. We did find that the overall seasonal distribution of excluded cases differed significantly from those included in our final sample (*p* < 0.001). Those without a relevant antibiotic fill within 30 days had a higher proportion of cases diagnosed in the ‘medium’ and ‘low’ months compared to those in our final sample with a fill, who had more cases diagnosed in the traditional ‘high’ incidence, summer months. This proportion (59%) is similar to reported CDC surveillance data in which 54% of LD cases were diagnosed in these months, lending confidence that our sample with a relevant antibiotic fill within 30 days was more reflective of the overall population of newly incident LD cases [23].

While this may suggest that many of those we excluded were not new diagnoses, it is also possible that some were but their antibiotic treatment was filled elsewhere not captured in our claims data. Similarly, we may have underestimated cases or outpatient care visits resulting from utilization that doesn’t generate ICD data, such as phone calls or complementary/alternative care not covered by insurance. In general, while we attempted to include all types of outpatient visits that, based on our clinical experience, typically encompass evaluation of persistent symptoms following treatment for LD, our definition of ‘LD-relevant’ care was subjectively determined and may have ultimately been over or under-specific, affecting the magnitude of our findings. It would be informative to determine which specific types of specialty care are driving these increases in future studies that are better able to parse out and identify PTLD.

## Conclusions

We examined the impact of LD diagnosis on outpatient care utilization in a sample of patients from a Lyme-endemic state and found modest, statistically significant increases at the population level in the post-LD diagnosis period. These increases were most marked among adult women and in specialty care visits. As the number of LD cases, and subsequently the cumulative prevalence of persistent symptoms after treatment continues to increase, it will be crucial to understand both the individual and population-level impact of this condition on quality of life, care utilization, and cost. We hope that future studies will confirm these findings as well as seek to examine and understand their significance in the context of other post-acute infectious syndromes.

### Supplementary Information

**Additional file 1: Supplementary Table 1.** Sensitivity analysis only including members who contributed continuous data at each month over the 48-month study interval. Generalized linear mixed effects regression models with number of all Lyme disease-relevant outpatient visits as the outcome. Models were run among the overall sample [1], as well as among adult [2] and children [3] strata only. Additional models [4, 5, and 6] were run with a gender and Lyme disease diagnosis period (pre vs post) interaction term included.

## Data Availability

The data that support the findings of this study are available from Johns Hopkins HealthCare, LLC but restrictions apply to the availability of these data, which were used under permission for the current study and so are not publicly available. Data are available from the authors upon reasonable request and with permission of Johns Hopkins HealthCare, LLC.

## Abbreviations

LD: Lyme Disease
CDC: Centers for Disease Control and Prevention
PTLD: Post-Treatment Lyme Disease
EHP: Employer Health Programs
ICD: International Classification of Diseases
RR: Rate ratio
IQR: Interquartile range
