# Supplementary material for: Outpatient visits before and after Lyme disease diagnosis in a Maryland employer-based health plan
Source: BMC Health Serv Res. 2023 Aug 29;23:919. doi: 10.1186/s12913-023-09909-3 (PMC10466890; doi:10.1186/s12913-023-09909-3)
Supplement: Supplementary file 1 — Additional file 1: Supplementary Table 1. Sensitivity analysis only including members who contributed continuous data at each month over the 48-month study interval. Generalized linear mixed effects regression models with number of all Lyme disease-relevant outpatient visits as the outcome. Models were run among the overall sample [1], as well as among adult [2] and children [3] strata only. Additional models [4, 5, and 6] were run with a gender and Lyme disease diagnosis period (pre vs post) interaction term included. [file 12913_2023_9909_MOESM1_ESM.docx]

**Supplementary Table 1**. Sensitivity analysis only including members who contributed continuous data at each month over the 48-month study interval. Generalized linear mixed effects regression models with number of all Lyme disease-relevant outpatient visits as the outcome. Models were run on the overall sample [1], as well as on adult [2] and children [3] strata only. Additional models [4, 5, and 6] were run with a gender and Lyme disease diagnosis period (pre vs post) interaction term included.

|  | ***Overall Sample***  ***N=95^a^*** | | ***Children Only***  ***N=25 ^b^*** | | ***Adults Only***  ***N=70^b^*** | |
| --- | --- | --- | --- | --- | --- | --- |
| ***Models with no interaction terms*** | **Model 1** | | **Model 2** | | **Model 3** | |
|  | **RR**^c^ | **p-value** | **RR** | **p-value** | **RR** | **p-value** |
| Pre-Lyme disease diagnosis period | REF | REF | REF | REF | REF | REF |
| Post-Lyme disease diagnosis period | 1.42 [1.26, 1.59] | <0.001 | 1.23 [0.94, 1.63] | 0.138 | 1.44 [1.26, 1.65] | <0.001 |
| Men | REF | REF | REF | REF | REF | REF |
| Women | 1.69 [1.18, 2.42] | 0.004 | 1.29 [0.81, 2.04] | 0.283 | 1.93 [1.21, 3.09] | 0.006 |
| Age (10 years) | 1.09 [0.99, 1.20] | 0.084 | 1.21 [0.62, 2.34] | 0.576 | 1.17 [0.94, 1.47] | 0.166 |
| Winter | REF | REF | REF | REF | REF | REF |
| Spring | 1.06 [0.91, 1.24] | 0.450 | 1.15 [0.81, 1.63] | 0.434 | 1.04 [0.87, 1.24] | 0.673 |
| Summer | 1.17 [1.00, 1.37] | 0.051 | 1.19 [0.84, 1.70] | 0.324 | 1.16 [0.97, 1.39] | 0.098 |
| Fall | 1.02 [0.87, 1.19] | 0.816 | 1.48 [1.06, 2.07] | 0.023 | 0.91 [0.76, 1.09] | 0.319 |
| ***Models with a gender interaction term*** | **Model 4** | | **Model 5** | | **Model 6** | |
|  | **RR** | **p-value** | **RR** | **p-value** | **RR** | **p-value** |
| Pre-Lyme disease diagnosis period | REF | REF | REF | REF | REF | REF |
| Post-Lyme disease diagnosis period | 1.20 [1.01, 1.44] | 0.044 | 1.27 [0.90, 1.80] | 0.175 | 1.15 [0.92, 1.44] | 0.209 |
| Men | REF | REF | REF | REF | REF | REF |
| Women | 1.46 [1.00, 2.14] | 0.049 | 1.34 [0.79, 2.27] | 0.281 | 1.60 [0.98, 2.61] | 0.058 |
| Women: Post-Lyme disease diagnosis period (interaction) | 1.31 [1.04, 1.65] | 0.023 | 0.93 [0.57, 1.51] | 0.773 | 1.41 [1.08, 1.85] | 0.012 |
| Age (10 years) | 1.09 [0.99, 1.20] | 0.086 | 1.21 [0.62, 2.34] | 0.575 | 1.17 [0.93, 1.46] | 0.172 |
| Winter | REF | REF | REF | REF | REF | REF |
| Spring | 1.06 [0.90, 1.24] | 0.477 | 1.15 [0.81, 1.63] | 0.430 | 1.04 [0.87, 1.24] | 0.698 |
| Summer | 1.17 [1.00, 1.37] | 0.051 | 1.19 [0.84, 1.70] | 0.323 | 1.16 [0.97, 1.38] | 0.099 |
| Fall | 1.02 [0.87, 1.20] | 0.801 | 1.48 [1.06, 2.07] | 0.022 | 0.91 [0.76, 1.09] | 0.332 |

***^a^***N: number of unique members.

^b^For multi-variate analyses, we defined adult status based on age at the last available month of data to keep adult status unique for each member. Therefore, the numbers of children and adults do not exactly match Table 1, in which adult status was defined based on age at the time of Lyme disease diagnosis; there were 5 members who were < 18 years of age (children) at the time of Lyme disease diagnosis who were subsequently ≥ 18 years of age (adults) when they contributed their last month of data.

*^c^RR: rate ratio. All but the interaction terms are rate ratios. The interaction terms in Models 4, 5, and 6 are ratios of rate ratios.*
